# Changes in physical activity and screen time related to psychological well-being in early adolescence: findings from longitudinal study ELANA

**DOI:** 10.1186/s12889-016-3606-8

**Published:** 2016-09-15

**Authors:** Viviane S. Straatmann, Aldair J. Oliveira, Mikael Rostila, Claudia S. Lopes

**Affiliations:** 1 Department of Epidemiology, State University of Rio de Janeiro (UERJ), Rio de Janeiro, Brazil; 2 Laboratory of Social Dimensions Applied to Physical Activity and Sport (LABSAFE), UFRRJ, Seropédica, Brazil; 3 Center for Health and Equity Studies (CHESS), Stockholm, Sweden

**Keywords:** Psychological well- being, Physical activity, Screen time, Adolescents, Longitudinal study

## Abstract

**Background:**

Psychological well-being influences health behaviours differently in adolescent boys and girls. We evaluated the role of psychological well-being in early adolescence in the onset and persistence of insufficient physical activity and exceeding recommended screen time, depending on gender.

**Methods:**

This work derives from a cohort study called Longitudinal Study of Adolescent Nutritional Assessment conducted among elementary school students from two public and four private schools in Rio de Janeiro, Brazil from 2010–2013. We analysed data from 2010 and 2012 from 526 adolescents. Physical activity was evaluated using the International Physical Activity Questionnaire. Those who performed less than 60 min per day of moderate to vigorous physical activity (MVPA) were classified as insufficiently active. Screen time was evaluated based on daily time spent in front of television, video games, and computers. Those who had 4 h or more screen time per day were classified as exceeding the recommended time. Psychological well-being was assessed using the psychological domain of the KIDSCREEN 27 questionnaire. Linear regression was used to estimate coefficient (β) and *r*^2^ values for continuous variables. Relative risks (RR) and confidence intervals (95 % CI) for onset and persistence of insufficient activity and exceeding recommended screen time were estimated with Poisson regression models.

**Results:**

Among girls, linear regression analyses showed a significant inverse association between psychological well-being and screen minutes per day at T2 (*r*^2^ = 0.049/β = −3.81 (95 % CI −7.0, −0.9)), as well as an association between poor psychological well-being and onset of exceeding recommended screen time in categorical analyses (RR crude: 1.3; CI 95 % 1.1, 1.7; RR adjusted: 1.3; CI 95 % 1.0, 1.6). For boys, an association was found between psychological well-being and onset of insufficient activity 2 years later (RR crude: 1.3; CI 95 % 1.2, 1.4; RR adjusted: 1.2; CI 95 % 1.1, 1.4).

**Conclusion:**

Adolescence is crucial for the development of unhealthy behaviours related to psychological well-being status in the context of a middle-income country. Gender differences are important because poor psychological well-being seems to affect sedentary behaviour in girls more than in boys, and predicts insufficient activity among boys.

## Background

Early adolescence is a critical period for the development and establishment of behaviours and attitudes [1]. Approximately 70 % of young people live in developing countries where complex economic, social, political, and environmental contexts create a wide range of challenges for adolescents to surmount on their journey to adulthood. Worldwide, the proportion of 13–15-year-olds that spend less than 60 min on moderate and vigorous physical activities (MVPA) per day, and spend more than 2 h a day in front of television (TV), is 80.3 % and about 65 %, respectively [2]. In Brazil, the *National Survey of Health of the School* (PeNSE) (2012) [3], reported that 70 % of schoolchildren do not meet the recommended level of MVPA and about 80 % spent excessive time in front of TV [4].

Collectively, the research suggests that MVPA for at least 60 min per day would help children and youth maintain a healthy profile. The documented health benefits include increased physical fitness (both cardiorespiratory fitness and muscular strength), reduced body fat, favourable cardiovascular and metabolic disease risk profiles, enhanced bone health, and reduced symptoms of depression [5]. The decrease of MVPA during adolescence is a consistent finding in the literature, being greater in girls at the beginning of adolescence [6], concomitant with an increasing amount of media exposure and sedentary behaviours [7]. While declines in MVPA have been repeatedly documented, less research attention has been directed toward understanding determinants leading to adolescent disengagement from activity and healthier behaviours. Furthermore, persistence of these unhealthy behaviours through adolescence and early adulthood is detrimental to health and well-being, and it has been shown to be associated with a less healthy lifestyle course [8, 9].

Research on determinants of physical activity has burgeoned in the past two decades, but has mostly focused on socio-demographic factors in high-income countries [10]. Given the estimates that approximately one in five young people under the age of 18 experience some form of developmental, emotional, or behavioural problems, added to the fact that neuropsychiatric disorders are a leading cause of health-related burden [11], there is a need to assess whether poor psychological well-being can influence young people to become less active and more sedentary or to persist with these behaviours during adolescence, in the context of a middle income country.

Psychological well-being of adolescents refers to being content with life and experiencing an abundance of positive emotions; when combined with the absence of psychological disorders, it is linked with the highest academic functioning, social skills and support, and physical health. For this reason, psychological well-being is very important for adolescence because it influences the development of a strong personality in the future, as well as influencing which life goals, values, direction, and purpose in life are selected [12].

Although the mechanisms and causal direction underlying the association between psychological well-being and inactive behaviour are still not clear, the following theories are cited: deficiency of serotonin and endorphins associated with decreases in hardiness; reduced stress reactivity; and related decreases in control, mastery, and self-efficacy to adapt and persist in activity [13]. Another particular factor that could lead to low physical activity is the psychological response accompanying the physical changes related to pubertal development [14, 15].

In addition, there is consistent evidence that there are differences between boys and girls in cognitive and behavioural coping at the beginning of adolescence [16]. Ryan and Connell [17] found that 8–12-year-old boys reported significantly more activities involving physical exercise than girls, whereas girls reported more social support and emotional behaviour than boys. Thus, gender represents a meaningful variable in research involving the coping strategies of young adolescents [18].

A growing number of studies confirm the power of well-being scales to predict outcomes related to health and lifestyle. Much recent research has found generally positive associations between psychological well-being and MVPA, as well as negative associations with sedentary behaviours [19–21]. Rothon et al. [22] found that more than 60 % of adolescents with mood disorders and 40 % of those with anxiety disorders reported low levels of MVPA. However, most of these studies were conducted in the context of European and Northern American countries with cross-sectional designs, not allowing a more robust discussion about the direction of association between variables and of causality. Thus, longitudinal designs, with more than one measurement of physical activity and sedentary behaviour outcomes, may yield more powerful conclusions.

In view of the above information, the aim of this study was to evaluate whether initial poor psychological well-being in early adolescence would be associated with the onset and persistence of insufficient activity and exceeding recommended screen time assessed 2 years later, and whether these associations would differ depending on gender.

## Methods

### Study design

The Longitudinal Study of Adolescent Nutritional Assessment (ELANA) is a longitudinal study following adolescents from two cohorts, a middle school cohort (age at baseline: 10 to 15 years) and a high school cohort (age at baseline: 13.5 to 19 years), at two public and four private schools from the metropolitan region of Rio de Janeiro, Brazil. Regarding the middle school cohort, during the follow-up period adolescents were assessed four times, during 2010–2013. The main aim of the ELANA was to examine changes in anthropometric indicators and body composition, as well as to study the influence of factors such as dietary intake, physical activity, alcohol and tobacco, and socioeconomic and psychosocial conditions on inadequate development and nutritional status.

This study included data from the middle school cohort at baseline, 2010 (Time1 = T1), and 2012 (Time 2 = T2). At T1, a self-report questionnaire was administered for the study of psychological well-being, physical activity, sedentary behaviour, and socioeconomic variables among respondents. Physical activity and sedentary behaviour measurements were repeated at T2.

This research was conducted according to the guidelines laid down in the Declaration of Helsinki and all procedures involving human subjects were approved before the start of the examinations by the Ethics Committee in Research of the Institute of Social Medicine of the State University of Rio de Janeiro (certificate number 0020.0.259.000-09). Written informed consent was obtained from adolescents’ legal guardians.

### Study sample

Out of 946 adolescents available, 888 met the eligibility criteria of not having a physical or mental condition preventing the completion of questionnaires and/or not being pregnant or lactating at the time. Of the 888 eligible adolescents, 32 (3.6 %) refused to participate, 46 (5.2 %) did not have parental consent, and four (0.45 %) were without birth date information, leading to a population study of 810 students (91.2 %) at baseline.

**Fig. 1:**
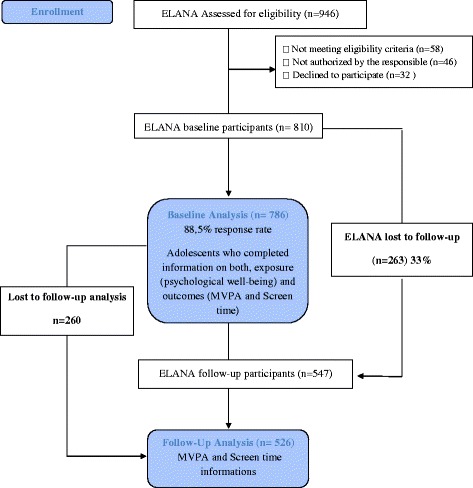
Flow diagram ELANA 2010 and 2012

For the present study, 786 adolescents completed information on both psychological well-being and outcomes (physical activity and sedentary behaviour) at T1, corresponding to a response rate of 88.5 %. At T2 we retrieved information on physical activity and sedentary behaviour for 526 adolescents, representing a 33 % decrease in participation. The data used in the current study is restricted to those adolescents who participated in both waves and had full information on all study variables (*n* = 526). Information about those who did not answer outcome questions from T1 to T2 can be found in Appendix (Fig. 1).

### Data collection

#### Outcomes

##### Physical activity

Physical activity was assessed with the self-reported ‘International Physical Activity Questionnaire (IPAQ)’ at T1 and T2. A short version of the IPAQ with eight open questions was used allowing us to estimate time spent in different physical activities (walking and physical efforts of moderate and vigorous intensity) and physical inactivity (sitting position) during the last 7 days.

This questionnaire was validated for Brazilian adolescents older than 14 years old by Guedes et al. [23]. When blank answers and unusual values for responses (e.g. adolescent told perform 5 h of moderate to intense physical activity per day) were detected, the research supervisor returned to the student in order to check the coherence of the response to avoid loss of information and error in the classification of the individual. The duration (minutes) of moderate and vigorous physical activity (MVPA) per day reported by adolescents was used based on the guidelines for data processing and analyses of the IPAQ [24]. The adolescents who did not meet the recommendation of 60 min a day of MVPA were classified as ‘insufficiently active’ [5].

##### Sedentary behaviour (Screen time)

The time watching television (TV) and using videogames/computers, designated ‘screen time’, was determined by a self-reported questionnaire comprising two questions at T1 and T2 [25]. The first question was *‘How many days do you watch TV and videogames/computers per week?’* The answers were categorized using a five-point scale for TV and videogames/computers: (1) almost never or never, (2) 1 to 2 times per week, (3) 3 to 4 times per week, (4) 5 to 6 times per week, and (5) every day. The intervals were converted into days per week as follows: category 1 = no days (almost never or never), 2 = 1.5 days, 3 = 3.5 days, 4 = 5.5 days, 5 = 7 days.

The second question was *‘In general, how many hours do you usually spend watching TV and videogames/computers per day?’.* Average daily time in minutes was calculated by multiplying ‘hours per day’ by ‘days per week’ for TV and videogames/computers applying this formula: [(days per week)*(hours per day)]*60/7, utilized as a continuous variable. Those who spent more than 4 h of screen time per day were classified as ‘exceeding recommended screen time’.

#### Independent variables

##### Psychological well-being

Psychological well-being was evaluated at T1 through the KIDSCREEN, a self-report questionnaire, designed to evaluate generic health related quality of life of children and adolescents [26]. The KIDSCREEN-27 was developed as a short version of the full version (KIDSCREEN-52) and comprises five dimensions of Rasch scales: Physical Well-Being (5 items), Psychological Well-Being (7 items), Autonomy and Parents (7 items), Peers and Social Support (4 items), and School Environment (4 items). The sub-dimension of psychological well-being used for this study is related to positive or negative attributes regarding emotional symptoms, life satisfaction, as well as feelings of sadness and loneliness. Whereas all the items in a particular area are indicators of a single one-dimensional latent trait, it is reasonable to use each domain in isolation for individual diagnostic interpretations targeted to an area of particular interest [27]. The questionnaire posed questions regarding the last week and for each item five options were provided on a 5-point Likert scale from 1 = never to 5 = always or from 1 = not at all to 5 = extremely. From the raw scores, t values were calculated, based on the Rasch model [27]; lower scores reflected worse psychological well-being. Scores of psychological well-being were dichotomized into ‘poor’ or ‘good’ psychological well-being. The ‘poor’ category was composed of individuals with scores of psychological well-being up to the 10^th^ percentile, and the ‘good’ category of those classified into percentiles higher than the 10^th^, forming the reference category.

#### Covariates

The following covariates were tested in the analysis: age, type of school, assets indicator, sexual maturation, and Body Mass Index (BMI). We included age in models as a continuous variable. We used type of school (private or public) as a *proxy* for socioeconomic status, because in Brazil, socio-demographic and economic characteristics directly impact selection of the type of school.

The economic characteristics of the families of adolescents were also represented by an indicator constructed from information on ownership of durable assets in the home according to the methodology used by Szwarcwald et al. [28], calculated with a weighted equation considering presence or absence of colour TV, VCR or DVD player, radio, bathroom, automotive, washing machine, refrigerator, and freezer (independent appliance or part of a duplex refrigerator). We used a dichotomized form of the assets indicator variable, once smoothing via polynomial split showed a clear difference between the first and the other quintiles on the psychological well-being T-scores, described by Agathão [29], in this sample. There was a marked slope from the first to the second quintile, followed by a plateau, justifying the category of ‘less asset ownership’ for the first quintile, as well as the ‘more asset ownership’ category for the other quintiles [29].

A validated self-assessment questionnaire [30] was employed to measure sexual maturation by using gender-specific line drawings of the stages of puberty [31]. This assessment was performed as a categorical variable (pre-pubescent, early spurt, maximum speed peak, and slowing growth). The BMI (kg/m^2^) was calculated based on direct measurements of height and weight according to Gordon and colleagues’ protocol [32]. BMI was included in the models as a dichotomous variable, comprising low/normal weight (BMI <25) and overweight/obese (BMI ≥ 25) [33].

### Data analysis

Descriptive statistics were provided by calculating the means and 95 % confidence intervals (95 % CI) for the continuous variables and the frequencies for count variables. Comparisons between genders were performed with independent samples t-tests and Chi Square Tests. Paired t-tests were applied to evaluate differences between means of minutes of MVPA and screen time from T1 to T2.

For continuous variables, two models were developed considering the following outcomes: 1) minutes per day of MVPA at T2, and 2) screen time minutes per day at T2. The variable minutes of MVPA at T1 was included in the model of MVPA outcomes (1) and screen time minutes per day at T1 for the screen time model at T2 (2) to adjust for baseline levels of these variables. Other possible confounders (age, sexual maturation, BMI, assets indicator, type of school) were tested for both models and only those with statistical significance (*p* < 0.20), were included in the final models, following a stepwise method to select the variables. As the most important variable for this criterion is not necessarily statistically significant, we imposed an upper limit on the *p*-values (the usual values are in the range [0.15–0.25]) for these variables, so that we selected candidates important in principle to the analysis.

Due the fact that the distribution of the variables was not normal (Kolmogorov-Smirnov test *p* < 0.05), the Log of the background variables and outcomes were used to perform linear regression procedures to determine the coefficients (β) and *r*^2^ values. Inverse Log values of β and *r*^2^ were presented to allow easier interpretation of the results.

Categorical analyses were also performed using the outcomes of insufficient activity and exceeding recommended screen time, with both analyses using psychological well-being at T1 as the independent variable. Two different approaches were used for each outcome. The first approach evaluated the onset of insufficient activity and exceeding recommended screen time at T2 (those who were sufficiently active or stayed within the recommended screen time at T1 who became inactive or exceeded recommended screen time per day at T2). For the onset analyses those who were insufficiently active and those who exceeded the recommended screen time at T1 were not considered. The second approach assessed persistence in being insufficiently active and exceeding recommended screen time at T2 (those who were insufficiently active or who exceeded the recommended screen time at T1 and maintained the same behaviour at T2). Only those were insufficiently active and exceeded the recommended screen time at T1 were considered for persistence analyses. Crude models for MVPA (onset and persistence) included the variable of minutes per day of MVPA at T1; screen time models (onset and persistence) added screen time minutes at T1, and age to adjust for these variables. The adjusted models tested the same variables and criteria as the continuous models.

To estimate the relative risks (RR) and their 95 % CI, we ran Poisson regression models using the sandwich estimator procedure. All statistical analyses were stratified by gender. Statistical analyses were performed using SPSS version 22.0 (SPSS Inc., Chicago, IL, USA).

## Results

The mean age was 11.0 (10.9–11.1) years old for girls and 11.2 (11.0–11.3) years old for boys at T1. A summary of the descriptive statistics is shown in Tables 1.  Statistically significant differences were observed in the comparison between the means of minutes of MVPA from T1 to T2 for both girls (T1 = 43.9 (38.4–49.5); T2 = 29.5 (24.7–34.3); *p* < 0.001) and boys (T1 = 66.0 (58.7–73.3); T2 = 42.1 (37.2–46.9); *p* < 0.001), and also between the means of screen time minutes per day from T1 to T2 in both genders (boys: T1 = 320.9 (293.6–348.2). T2 = 355.5 (326.3–384.7), *p* = 0.026; girls: T1 = 301.1 (271.1–331.1), T2 = 381.1 (348.9–413.3), *p* < 0.001) (Table 1).

**Table 1:** Characterization of sample by sex: middle school cohort, ELANA study, 2010 and (2012)

Variables	Male *N* = 277	Female *N* = 249	*P* value
T1 (2010)			
Age (CI 95 %)	11.2 (11.0–11.3)	11.0 (10.9–11.1)	*0.037* ^a^
Sexual maturation (%)			
Pre-pubescent	5.8	6.4	*<0.001* ^b^
Early spurt	73.1	28.9	
Maximum speed peak	16.3	47.0	
Slowing growth	4.4	17.7	
Type of school (%)			
Public	30.7	30.9	0.953^b^
Private	69.3	69.1	
Assets Indicator (%)			
Less asset ownership	25.3	12.7	*<0.001* ^b^
More asset ownership	74.7	87.3	
BMI (Kg/m^2^)			
Mean (CI 95 %)	20.6 (20.1–21.2)	20.2 (19.7–20.7)	0.265^a^
Normal (%)	82.4	87.8	
Overweight/obesity (%)	17.6	12.2	0.090^b^
MVPA (min/day)			
Mean (CI 95 %)	66.0(58.7–73.3)	43.9(38.4–49.5)	<0.001^a^
> 60 min/day^c^ (%)	36.5	22.5	
≤ 60 min/day ((%)	63.5	77.5	*<0.001* ^b^
Screen time (min/day)			
Mean (CI 95 %)	320.9 (293.6–348.2)	301.1(271.1–331.1)	0.337^a^
≤ 4 h/day^c^ (%)	48.0	53.6	
> 4 h/day (%)	52.0	46.4	0.199^b^
Psychological well-being			
Mean (CI 95 %)	49.5 (47.5–50.1)	50.2(48.8–51.4)	0.405^a^
Poor (%)	12.3	13.3	
Good (%)^c^	87.7	86.7	0.737^b^
T2 (2012)			
MVPA (min/day)			
Mean (CI 95 %)	42.1(37.2–46.9)	29.5(24.7–34.3)	*<0.001* ^a^
> 60 min/day^c^ (%)	26.0	13.3	
≤ 60 min/day ((%)	74.0	86.7	*<0.001* ^b^
Screen time (min/day)			
Mean (CI 95 %)	355.5(326.3–384.7)^d^	381.1(348.9–413.3)^d^	0.245^a^
≤ 4 h/day^c^ (%)	43.7	39.8	
> 4 h/day (%)	56.3	60.2	0.362^b^

*CI 95 %* confidence interval 95 %, *BMI* body mass index, *MVPA* moderate and vigorous physical activity; ^a^T test for independent sample by gender; ^b^Qui Square test by gender; ^c^Reference category- Highers scores of psychological well-being; ^d^Significant difference between means over time (*p* < 0,05)-Pared T test

Among girls who were sufficiently active (*N* = 56) and did not exceed the recommended screen time (*N* = 119) at T1, 67.8 % (N = 38) became insufficiently active and 38.6 % (*N* = 46) exceeded recommended screen time minutes at T2. Females who remained insufficiently active (T1: *N* = 192) and who exceeded recommended screen time (T1: *N* = 128) from T1 to T2 were 87.5 % (*N* = 168) and 53.1 % (*N* = 68), respectively. Among boys who were sufficiently active (*N* = 100) and did not exceed the recommended screen time (*N* = 133) at T1, 74 % (*N* = 74) became insufficiently active and 33.8 % (*N* = 45) exceeded the recommended screen time at T2. Persistence of insufficient activity (T1: *N* = 172) and exceeding recommended screen time (T1: *N* = 137) in males was 73 % (*N* = 126) and 63.5 % (*N* = 87), respectively.

The linear regression analyses showed a significant inverse association between psychological well-being scores and screen minutes per day at T2 among girls (*r*^2^ = 0.049/β = −3.81(95 % CI −7.0, −0.9)). The inclusion of screen time at T1, significant in the model (β = 0.15 (95 % CI 0.02, 0.3)), did not alter the significance of the final model. Models based on screen time at T2, adjusted for screen minutes per day at T1 explained 4.9 % of the variance in screen time in girls, and 16 % among boys (in a non-significant model). There was no association between psychological well-being scores and minutes per day with MVPA at T2 for both genders, and the models explained 1.1–1.8 % of the variance of these variables (Table 2).

**Table 2:** Coefficient (β) of linear regression, *r* ^2^ and *p* value for the association between MVPA and screen time with psychological well-being by gender. ELANA study, 2010 and (2012)

Variables	Psychological well-being					
	Males			Females		
	B	*r* ^2^	CI 95 %	B	*r* ^2^	CI 95 %
MVPA at 2012 (min/day)	0.27	0.018	−0.2–0.7	0.22	0.011	−0.2–0.7
MVPA at 2010 (min/day)	1.0		−0.8–0.8	0.69		−0.04–0.7
Screen Time at 2010 (min/day)	0.02		0.0–0.04	-		-
Screen Time at 2012 (min/day)	−0.44	0.161	−3.3–2.4	−3.81	0.049	−7.0- −0.9
Screen Time at 2010 (min/day)	0.36		0.2–0.5	0.15		0.02–0.3
BMI (Kg/m^2^)	9.10		2.6–15.5	-		-

*β* coefficient of linear regression, *r* ^*2*^ coefficient of determination, *MVPA* moderate and vigorous physical activity, *BMI*, body mass index, *CI* confidence interval

For those with poorer psychological well-being, the risk of becoming insufficiently active was statistically significant, for both genders, on crude analyses (boy: RR crude 1.3; 95 % CI 1.2, 1.4/ RR adjusted 1.2; 95 % CI 1.1, 1.4; girls: RR crude 1.2; 95 % CI 1.1, 1.3). Regarding the association between the onset of exceeding recommended screen time and psychological well-being, statistical differences were observed among girls in crude (RR 1.3; 95 % CI 1.1, 1.7), as well as in adjusted models (RR 1.3; 95 % CI 95 % 1.0, 1.6). No associations were demonstrated between persistence of both types of undesired behaviour and psychological well-being in boys and girls (Tables 3 and 4).

**Table 3:** Relative risks (RR) and 95 % confidence intervals (95 % CI) of exposure to psychological well-being at T1 (2010) - tertiles and frequencies of onset and persistence of insufficiently active by gender. ELANA Study 2010 and 2012

	Onset of insufficiently active				Persistence of insufficiently active			
Psychological well-being	Not became	Became			Not persist	Persist		
	N (%)	N (%)	Crude RR (95 % CI)	Adjusted RR^a^ (95 % CI)	N (%)	N (%)	Crude RR (95 % CI)	Adjusted RR^b^ (95 % CI)
Male								
Poor	0 (-)	6 (5.9)	1.3 (1.2–1.4)	1.2 (1.1–1.4)	7 (4.0)	21 (11,9)	1.0 (0.8–1.2)	1.0 (0.9–1.2)
Good^1^	26 (25.7)	69 (68.3)	1.0 -	1.0 -	39 (22.2)	109 (61.9)	1.0 -	1.0 -
Female								
Poor	0 (-)	4 (7.1)	1.2 (1.1–1.3)	1.1 (0.9–1.4)	3 (1.6)	26 (13.5)	1.0 (0.9–1.1)	1.0 (0.9–1.2)
Good^1^	9 (16.1)	43 (76.8)	1.0 -	1.0 -	21 (10.9)	143 (74.1)	1.0 -	1.0 -

^a^Adjusted for age, MVPA at T1, type of school and screen time at T1 for male; age, MVPA at T1, BMI and screen time at T1 for girls; ^b^Adjusted for age, MVPA at T1, BMI, type of school and screen time at T1 for male; age, MVPA at T1, assets indicator and screen time at T1 for female; ^1^Reference category

**Table 4:** Relative risks (RR) and 95 % confidence intervals (95 % CI) of exposure to psychological well-being at T1 - tertiles and frequencies of onset and persistence of screen time by gender. ELANA Study 2010 and 2012

	Onset of Screen Time				Persistence of Screen Time			
Psychological well-being	Not became	Became			Not persist	Persist		
	N (%)	N (%)	Crude RR (95 % CI)	Adjusted RR^a^ (95 % CI)	N (%)	N (%)	Crude RR (95 % CI)	Adjusted RR^b^ (95 % CI)
Male								
Poor	8 (6.1)	9 (6.8)	1.1 (0.9–1.5)	1.1 (0.8–1.5)	8 (5.6)	9 (6.3)	0.8 (0.6–1.1)	0.8 (0.7–1.1)
Good^1^	69 (52.3)	46 (34.8)	1.0 -	1.0 -	34 (23.8)	92 (64.3)	1.0 -	1.0 -
Female								
Poor	10 (7.5)	3 (2.3)	1.3 (1.1–1.7)	1.3 (1.0–1.6)	6 (5.2)	14 (12.2)	1.0 (0.8–1.3)	1.0 (0.8–1.2)
Good^1^	61 (45.9)	59 (44.4)	1.0 -	1.0 -	31 (27.0)	64 (55.7)	1.0 -	1.0 -

^a^Adjusted for age, screen time at T1, BMI, type of school and MVPA at T1 for male; age, screen time at T1 assets indicator, sexual maturation and MVPA at T1 females; ^b^Adjusted for age, screen time at T1, assets indicator, BMI and MVPA at T1 for male; age, screen time at T1, type of school and MVPA at T1 for female; ^1^Reference category

## Discussion

This study examined the relationship between psychological well-being at baseline and amount of MVPA and screen time 2 years later, evaluating the onset and persistence of insufficient activity and exceeding recommended screen time in the context of a middle-income country. We also investigated whether these relationships differed between genders.

Marked declines are evident among youth, with surveys showing steadily decreasing proportions meeting minimum recommended levels of self-reported physical activity [34]. In addition, steep age-related declines are apparent in both cross-sectional [35, 36] and longitudinal studies among adolescents and pre-adolescents [37, 38], being in line with the results from the present study.

Leighton and Swerisson [39], who examined students’ perceived changes in physical activity, found 42 % of students reported a relative decline from high school to university. Another study also observed that one third of students declined into insufficient activity during the transition to university [40]. Our sample with children at the beginning of adolescence has shown higher percentages concerning the decline of MVPA per day in boys (74 %) and girls (67.8 %) indicating an important change of behaviours during this phase, in this social economic context target.

Regarding results about physical activities changes in the context of low/middle income countries, as in the present study, the first prospective data were from Brazil. The authors found that boys increased their leisure-time physical activity level over the 4 years (mean 75 min/week; 95 % CI 49, 100), whereas a decrease was observed among girls (mean −42 min/week; 95 % CI −57, −28) [41].

We also find significant differences related to screen time over 2 years, in line with a longitudinal study of American girls aged between 12 and 14 years old that showed an increase from 7.7 to 8.5 h per day in sedentary behaviour [42]. Mitchell and colleges [43] also found significant changes over time related to sedentary behaviour measured objectively in both genders, evaluated in the United Kingdom at ages 12, 14, and 16. One explanation for the significant increase of sedentary behaviour, especially screen time among adolescents, could be that the habit of watching TV has always had a significant social impact on the lives of children and adolescents [44]. Additionally, it should be noted that in recent years the time spent in sedentary activities among adolescents has increased largely due to excessive hours spent on computers, tablet devices, interactive games, and social media [45].

Our results did not verify an association between psychological well-being and MVPA for both genders, when considering the models with continuous variables. However, considering the categorical approach, poorer psychological well-being was associated with onset of insufficient activity in boys, and in girls only in the crude model. The literature has demonstrated conflicting results regarding this relationship. A study performed by Rothon et al. [22] with adolescents from East London indicated no significant association between changes in level of physical activity and odds of depressive symptoms at follow-up. Additionally, there was no evidence in this study for reverse causality; participants who became depressed between baseline and follow-up did not have higher odds of changing their physical activity levels.

In contrast, Bray and Born [41] found that adolescents who self-reported becoming insufficiently active during the first 2 months of transition between the end of adolescence to adulthood life, also reported lower levels of vigour and higher levels of fatigue when compared with those who had continued to be active 2 months after baseline. These researchers also published results from the same sample referring to 7 months after baseline, and insufficiently active students also reported lower levels of psychological well-being at the beginning of the study [8].

Another issue to consider that may justify controversy results in the literature is that some individual and contextual aspects related to MVPA could cause different coping strategies to deal with psychological problems. Research conducted in the Nordic context with Finnish adolescents (16 and 18 years old at baseline and follow up, respectively) showed that among girls who had psychosocial problems at baseline, these problems are simultaneously associated with a higher level of MVPA [46]. A systematic search indicated evidence that habitually active individuals are positively impacted by psychological stress, increasing their level of physical activity, and those at the beginning stages of practice tend to exercise less. Consequently, psychological stress may have a differential impact on exercise adoption, maintenance, and relapse [47].

Regarding psychological well-being and screen time, the literature is more consistent than for MVPA. We found associations between low psychological well-being and the onset of exceeding the recommended screen time among girls. In girls, psychological well-being seems to have more of an effect in determining sedentary behaviours. Among females, strategies to regulate emotions, particularly in early adolescence, are less likely to use distraction/recreation involving corporal movements, such as physical activity and aggression than their male counterparts [48]. A systematic review of the associations between health indicators and screen-based time among adolescent girls suggested a negative relationship between psychological well-being and screen time; however, more longitudinal studies are necessary [49]. One possible explanation of the underlying causes is that adolescent girls with specific symptoms of depression (e.g. feeling tired; finding it hard to initiate activities) may withdraw from social activities, preferring more solitary pursuits such as screen technologies over time [49]. Hume et al. [50] reported that Australian girls (14.4 years ± 0.61) with depressive symptoms in 2004 reported significantly higher TV, video, and DVD viewing in 2006. Another longitudinal study conducted in the Netherlands with 663 students (ages 12 to 15 years) observed that girls who reported more loneliness were particularly vulnerable to the onset of compulsive internet use 6 months later [51].

Currently, it is common to see adolescents of both genders playing videogames, using tablets/computers, and accessing social media in Brazil; however, the use of these types of screen technology among girls seems to be a more recent phenomenon in Brazil [52]. Perhaps for this reason, our results have shown associations among girls only when we consider the outcome 2 years later, not the persistence of this behaviour. Another economic issue to be discussed is that Brazil is a middle-income country with high taxes for media technologies and imported products, thereby tending to receive and incorporate novel technologies later than developed countries [52].

To the best of the authors’ knowledge, the current work is the first to evaluate the role of psychological well-being over time in MVPA and screen time in a sample of adolescents in Brazil. The longitudinal design allowed for new interpretations on the subject, reducing the possibility of reversed causality, which is an important strength of this study.

A few limitations of this study should be taken into account. Firstly, our results are based on self-reported information about physical activity and screen time. Self-report questionnaires present some limitations regarding the ability of adolescents to remember, interpret, and quantify physical activity [53, 54]. Despite recommendations that research combine the strengths of both self-report and objective measures to provide new insights into the benefits of physical activity and how to implement successful interventions, the majority of public health physical activity guidelines are based on self-report [55].

Secondly, the use of the IPAQ for adolescents younger than 14 years could be a limitation, since the validation study in Brazil suggests its application for adolescents older than 14 years [24]. Despite a plethora of self- or proxy-reported measures of physical activity for younger people, there is no internationally accepted standard for the measurement of physical activity in adolescents younger than 14 years old. One of the additional challenges is that variations in scoring protocols make it very difficult to compare results between studies [56]. Therefore, we decided to use the IPAQ for adolescents younger than 14 years, checking the coherence of the responses immediately after completion, minimizing biases and inconsistencies in the answers, as some others Brazilian studies have done [57–59]. Thirdly, a large proportion of individuals did not complete the measurement of physical activity and screen time at T2, reducing the sample by 33.1 %. Meanwhile, school changes by students are very common in Brazil, making it difficult to follow them, and these are probably not related to the outcomes or the other variables being investigated.

## Conclusion

Our study identified gender differences in the associations between psychological well-being and the outcome behaviours. The present study highlights the associations between initial poor psychological well-being and the onset of exceeding the recommended screen time 2 years later in girls. This information strengthens prior findings in the literature, and shows an important and new phenomenon among female adolescents in a middle-income context. Sedentary behaviour guidelines for youth may need to be regularly updated, reflecting the rapid technological changes and the significant engagement of adolescents in social media.

Specific strategies focusing on gender differences should be applied for prevention and intervention programs to increase physical activity levels, since prior poor psychological well-being in boys seems to have more negative effects on their MVPA, considering the criteria of public health guideline recommendations.

These results suggest important implications for public health, since the understanding of the determinants that affect physical activity and sedentary behaviours can assist in developing prevention initiatives focused on adolescent health and conduct of the work of school managers. Moreover, considering the consequences of unhealthy behaviors have for health throughout the life course, such as the development of chronic diseases, which, in turn, result in staggering costs to the public health management, evaluative and interventional epidemiological actions focused on preventive practices far beyond healing, become crucial in the current Brazilian health scenario.

Future studies should continue prioritizing longitudinal methodologies so that we can advance in knowledge of the physical activity epidemiology. Approaches that consider longer periods, covering all stages of adolescence and the joint use of direct measures of assessment of physical activity and sedentary (accelerometer) and questionnaires to obtain less biased and more complete information on these constructs. Modern and robust tools for data analysis should be explored in order to study complex scenarios more clearly.

## Appendix

### Characterization dropout from T1 to T2

About the missing between T1 (*N* = 786) and T2 (2012) (*N* = 526), among these 260 individuals was observed a difference in the distribution by gender (boys: 55.9 % vs girls: 44.1 %, *p* = 0.007), but there was no difference in the distribution by type of school (public: 51.3 % vs private: 48.7 %, *p* = 0.540). The average of minutes per day spent with screen time between those did not continued at T2 was 361.1 (328.3−349.3), 68.2 % of these did not meet the daily recommendations of PA and the average score of psychosocial being was 47.6 (40.2−53.1).

## Abbreviations

BMI: Body mass index
CI: Confidence interval
ELANA: Longitudinal study of adolescent nutritional assessment
IPAQ: International physical activity questionnaire
MVPA: Moderate and vigorous physical activity
PeNSE: *National Survey of Health of the School*
RR: Relative risk
T1: Time 1
T2: Time 2
TV: Television
